# The Association of Cognitive Behavioral Play Therapy Exposure With Self-Esteem and Self-Efficacy Among School-Age Children With Learning Disabilities: An Observational Study

**DOI:** 10.7759/cureus.118013

**Published:** 2026-10-16

**Authors:** Yamini Selvaraj, K Cheran, N Gowri, Gaoudam Nadarajane, Pradeep Pazare

**Affiliations:** 1 Department of Child Health Nursing, Vinayaka Mission's Research Foundation (Deemed to be University), Salem, IND; 2 Department of Anesthesiology, Vinayaka Mission's Medical College, Vinayaka Mission's Research Foundation (Deemed to be University), Karaikal, IND; 3 Department of Nursing, Chettinad College of Nursing, Chettinad Hospital and Research Institute, Chettinad Academy of Research and Education, Chettinad University, Kelambakkam, IND; 4 Department of Medical Surgical Nursing, Vinayaka Mission's College of Nursing, Vinayaka Mission's Research Foundation (Deemed to be University), Karaikal, IND; 5 Department of Pediatric Medicine, N. K. P. Salve Institute of Medical Sciences and Research Centre and Lata Mangeshkar Hospital, Nagpur, IND

**Keywords:** children, cognitive behavioural play therapy, general self-efficacy scale, learning disabilities, psychosocial functioning, rosenberg self-esteem scale, self-efficacy, self-esteem

## Abstract

Background and objective

Learning disabilities in children can affect their emotional and social functioning as well as academic performance and self-perception. Cognitive-behavioral play therapy (CBPT) is a therapeutic approach that may help address psychosocial difficulties in children. However, the evidence on the association between prior CBPT exposure and the self-esteem and self-efficacy of children with learning disabilities remains limited. This study examined the association between prior CBPT exposure and self-esteem and general self-efficacy among school-age children with learning disabilities.

Methods

This cross-sectional observational study included children aged 10-14 years who had been diagnosed with learning disabilities. Prior exposure to CBPT was determined based on information provided by the participants or their parents, as well as medical records. Participants were classified into three groups according to their previous CBPT exposure: 0 sessions, 1-11 sessions, and ≥ 12 sessions. The levels of self-esteem and general self-efficacy were assessed using the Rosenberg Self-Esteem Scale (RSES) and the General Self-Efficacy Scale (GSE). One-way analysis of variance (ANOVA) and Tukey’s post hoc test were used to compare differences among the three groups.

Results

Among the 125 participants, 50 (40.0%) had no previous CBPT exposure, 40 (32.0%) had completed 1-11 sessions, and 35 (28.0%) had completed ≥ 12 sessions. Mean RSES scores were 24.8 ± 3.8, 27.5 ± 3.8, and 29.8 ± 3.4, respectively, with a significant difference among the groups (p < 0.001). Mean GSE scores were 27.1 ± 4.7, 30.0 ± 4.6, and 32.9 ± 4.0, respectively (p < 0.001). The number of CBPT sessions was positively correlated with RSES scores (r = 0.48, p < 0.001) and GSE scores (r = 0.46, p < 0.001). In multivariable regression analysis, the number of CBPT sessions remained significantly associated with RSES scores (adjusted β = 0.28, 95% confidence interval (CI): 0.18-0.38; p < 0.001) and GSE scores (adjusted β = 0.34, 95% CI: 0.22-0.45; p < 0.001) after adjustment for relevant demographic and clinical variables.

Conclusions

Greater previous CBPT exposure was associated with higher self-esteem and general self-efficacy among children with learning disabilities. These findings suggest that children who had received CBPT previously had higher scores on both measures than those with less or no previous exposure. However, this relationship should be interpreted as correlational rather than causal because of the cross-sectional observational design. Further prospective and intervention studies are required to determine whether CBPT leads to improvements in self-esteem and self-efficacy among children in this group.

## Introduction

Learning disabilities can adversely affect a child's academic progress and are often accompanied by low self-esteem and difficulties with psychological adjustment, as parents and teachers have observed [1,2]. Self-efficacy is another important consideration among children and adolescents with learning disabilities. Evidence suggests that children with learning disabilities may have lower self-efficacy, which can undermine their confidence in managing academic tasks and interpersonal challenges [3,4]. These psychosocial difficulties can be particularly significant in the school setting, where repeated academic struggles and perceived failure may contribute to negative self-perceptions and feelings of incompetence [5].

In this study, learning disabilities refer to specific learning difficulties that affect the acquisition and use of academic skills, including reading, written expression, and mathematics. The World Health Organization’s International Classification of Diseases, 11th Revision (ICD-11), classifies this condition as developmental learning disorder (code 6A03), which is characterized by persistent and significant difficulties in academic skills that are substantially below age expectations and impair academic functioning [6]. These difficulties are not primarily attributable to intellectual developmental disorders, sensory impairments, inadequate educational opportunities, or insufficient proficiency in the language of instruction [6]. The American Psychiatric Association similarly classifies this condition as specific learning disorder in the Diagnostic and Statistical Manual of Mental Disorders, Fifth Edition, Text Revision (DSM-5-TR) [7].

Addressing psychosocial problems alongside academic difficulties is important for children with learning disabilities. Play therapy is a therapeutic approach used to address emotional and behavioral problems in children. Cognitive-behavioral play therapy (CBPT) integrates cognitive-behavioral principles with developmentally appropriate play activities to help children manage emotional and behavioral difficulties [8]. It may be particularly beneficial for children who may not respond well to traditional psychological approaches. Children with learning disabilities represent a heterogeneous population, with difficulties in reading, mathematics, written expression, or multiple academic domains. In addition, comorbid attention-deficit/hyperactivity disorder (ADHD) may contribute to further academic and psychosocial difficulties [9,10]. These clinical characteristics should be considered as potential covariates when examining the association between previous CBPT exposure and self-esteem and general self-efficacy.

Previous studies have investigated CBPT for a range of emotional, behavioral, and functional difficulties in children. A scoping review has mapped the use of play-based interventions among children with disabilities in low- and middle-income countries, highlighting their potential applications while identifying gaps in the evidence regarding implementation and impact [11]. However, the effectiveness of these interventions specifically for children with learning disabilities remains insufficiently established. A preliminary randomized controlled trial by Shayganfard et al. examined group CBPT for children with school refusal associated with anxiety and behavioral problems, focusing on anxiety-related school refusal and behavioral outcomes [12]. In a separate study, Vosoughi Kalantari et al. evaluated CBPT in students with dyslexia and reported improvements in behavioral adjustment and problem-solving skills following the intervention, with benefits maintained at follow-up [13].

Although these findings suggest that CBPT may be useful for addressing selected psychosocial and behavioral difficulties, the studies focused primarily on anxiety, behavior, adjustment, and problem-solving outcomes. The evidence on self-esteem and general self-efficacy among children with learning disabilities specifically remains limited. These outcomes are important because they reflect children's perceptions of their self-worth and their confidence in managing challenges. Therefore, the present study examined the association between prior CBPT exposure and self-esteem and general self-efficacy among children with learning disabilities.

Although earlier studies have examined CBPT in children with learning disabilities, including outcomes such as academic self-efficacy and self-esteem [14,15], the evidence on general self-efficacy and the relationship between the extent of prior CBPT exposure and psychosocial outcomes remains limited. In particular, it is unclear whether the number of previously completed CBPT sessions is associated with self-esteem and general self-efficacy after adjusting for relevant demographic and clinical factors. Therefore, this cross-sectional observational study examined the association between prior CBPT exposure and self-esteem and general self-efficacy among school-age children with learning disabilities. The study also examined whether these outcomes varied by the number of CBPT sessions completed previously.

## Materials and methods

Study design and setting

This cross-sectional observational study was conducted to assess the association between previous exposure to CBPT and self-esteem and self-efficacy among children with learning disabilities. The study was conducted at Vinayaka Mission's Medical College, Karaikal, Puducherry, from June 1, 2021 to May 31, 2022. Institutional Ethics Committee approval was obtained from the Institutional Ethics Committee of Vinayaka Mission's Medical College, Karaikal (IEC Approval No. VMMC/NURS/2021/56; approval date: May 27, 2021).

Recruitment and study procedures

Participants were recruited through the clinical services of Vinayaka Mission Medical College, Karaikal, Puducherry, between June 1, 2021, and May 31, 2022. Children aged 10-14 years with a documented diagnosis of learning disability were considered for participation. The research investigator explained the study opportunity and its purpose to eligible children and their parents or legal guardians in person. Eligibility was assessed using clinical records and clinical assessment, according to the study's selection criteria.

Socioeconomic status (SES) was assessed using the Modified Kuppuswamy Scale, which incorporates the educational level and occupational status of the head of the family and monthly family income. Scores for the three components were summed to obtain a composite score ranging from 3 to 29. Participants were classified into five socioeconomic categories: upper (Class I, 26-29), upper-middle (Class II, 16-25), lower-middle (Class III, 11-15), upper-lower (Class IV, 5-10), and lower (Class V, 3-4). The income component was interpreted according to the scale version applicable during the study period. For multivariable regression, the composite SES score was entered as a continuous predictor, with higher scores indicating higher SES.

Written informed consent was obtained from parents or legal guardians, and assent was obtained from participating children. Study procedures were conducted at the medical college. Demographic and clinical information was collected, and previous CBPT exposure was ascertained using the sources described in the study protocol, including medical records and participant or guardian reports. The number of previously completed CBPT sessions was recorded before study enrolment.

Self-esteem was assessed using the Rosenberg Self-Esteem Scale (RSES), and general self-efficacy was assessed using the General Self-Efficacy Scale (GSE) [16,17]. The questionnaires were administered in English, with assistance provided to children by the research investigator as the items were read aloud. Participants were classified according to their previous CBPT exposure as having received no sessions, 1-11 sessions, or ≥ 12 sessions. As this was a cross-sectional observational study, no intervention was assigned by the investigators.

Selection criteria

Children aged 10-14 years with a diagnosis of learning disability were included in the study. This age group was selected because children at this developmental stage are generally better able to understand questionnaire items, reflect on their self-perceptions, and report their feelings and beliefs about their abilities. These capacities are particularly relevant to assessing self-esteem and general self-efficacy, the primary outcomes of this study. Restricting the age range also helped establish a relatively comparable school-age population for examining the association between previous CBPT exposure and these psychosocial outcomes. Children with severe intellectual disability, severe neurological disorders, severe visual or hearing impairment, or an inability to understand and respond to the study questionnaires were excluded.

Diagnostic assessment

The diagnosis of learning disability was established by a clinical psychologist using the diagnostic criteria of the DSM. Comorbid ADHD was identified through assessment by a psychiatrist.

Sample size

The sample size was estimated using G*Power version 3.1.9.7 for a fixed-model multiple linear regression analysis, with an R² deviation from zero. The calculation assumed a medium effect size (f² = 0.15), a significance level of 0.05, 80% power, and 8 predictors. The calculated minimum sample size was 109 participants. The recruitment target was increased to 125 participants to allow for potentially incomplete or invalid observations. A total of 125 participants were included in the final analysis.

Data sources and variables

CBPT Exposure

Previous exposure to CBPT was ascertained from clinical records and reports provided by participants or their parents/guardians. Session counts were cross-checked against available clinical documentation. CBPT was delivered by a clinical psychologist and incorporated developmentally appropriate cognitive-behavioral and play-based activities, including emotion identification, recognition and modification of negative thoughts, problem-solving exercises, behavioral rehearsal, and reinforcement of adaptive coping strategies. 

The number of sessions completed before the study assessment was recorded for each participant. Among children with prior CBPT exposure, the mean number of completed sessions was 11.4 ± 7.1 (range: 1-24). The interval between the last session and study assessment was also recorded, with a mean interval of 4.6 ± 3.1 months (range: 1-12 months). Participants were classified into three groups based on prior exposure: no exposure (0 sessions), limited exposure (1-11 sessions), and higher exposure (≥ 12 sessions). Session count was also analyzed as a continuous variable in correlation and multivariable regression analyses. No intervention was assigned by the study investigators.

Outcome Measures

Self-esteem with regard to RSES was measured by 10 items, using 4-point scales [16]. All negatively framed statements were accordingly reversed, and item values were summed to consider the self-esteem scores. General self-efficacy was measured with the GSE by Schwarzer and Jerusalem [17]. GSE is a scale with 10 items scored on a scale of 1 to 4, where the total score can vary from 10 to 40 points. The GSE provides a general notion of self-efficacy as compared to more specific academic self-efficacy. Previous studies indicate the reliability of both GSE and RSES among adolescents [18,19].

Statistical analysis

Continuous variables were reported as mean ± standard deviation (SD), while categorical variables were expressed as frequencies and percentages. Differences in RSES and GSE scores across the three CBPT exposure groups were assessed using one-way analysis of variance (ANOVA). When the overall ANOVA was statistically significant, pairwise comparisons were performed using Tukey’s honestly significant difference test. Standardized mean differences were calculated as Hedges’ g, with corresponding 95% confidence intervals (CIs), to complement the raw mean differences and Tukey-adjusted p-values. Pearson correlation and multivariable linear regression were used to examine the associations between the number of previous CBPT sessions and the outcome scores. A two-sided p-value < 0.05 was considered statistically significant.

## Results

Participant characteristics

In this study, 125 individuals were recruited. Table 1 describes the demographic and clinical features of the research subjects, including age, sex, type of learning difficulties, diagnosis of ADHD, and prior exposure to CBPT.

**Table 1:** Demographic and clinical characteristics of the study population SD: standard deviation; ADHD: attention-deficit/hyperactivity disorder; CBPT: cognitive behavioral play therapy

Characteristic	Values
Total participants	125
Age (years), mean ± SD	12.5 ± 1.4
Sex, n (%)	
Male	70 (56.0)
Female	55 (44.0)
Learning-disability subtype, n (%)	
Reading-related	57 (45.6)
Mixed	41 (32.8)
Mathematics-related	17 (13.6)
Written-expression	10 (8.0)
Comorbid ADHD	36 (28.8)
Previous CBPT exposure, n (%)	
0 sessions	50 (40.0)
1-11 sessions	40 (32.0)
≥ 12 sessions	35 (28.0)
Any previous CBPT exposure	75 (60.0)

The mean age of the participants was 12.5 ± 1.4 years. Of the 125 participants, 70 (56.0%) were male, and 55 (44.0%) were female. Reading-related learning difficulty was the most common subtype, affecting 57 (45.6%) participants, followed by mixed learning difficulties in 41 (32.8%), mathematics-related difficulties in 17 (13.6%), and written-expression difficulties in 10 (8.0%) participants. Comorbid ADHD was present in 36 (28.8%) participants. Out of the 125 participants, 50 (40.0%) had no previous CBPT exposure, 40 (32.0%) had completed 1-11 sessions, and 35 (28.0%) had completed at least 12 sessions. Among the 75 participants with previous exposure, the mean number of completed sessions was 11.4 ± 7.1, with a range of 1-24 sessions. The mean interval between the last session and the study assessment was 4.6 ± 3.1 months (range: 1-12 months).

Self-esteem and general self-efficacy

Self-esteem and general self-efficacy differed significantly according to previous CBPT exposure, as shown in Table 2. The overall mean RSES score was 27.06 ± 4.20, while the overall mean GSE score was 29.65 ± 5.04.

**Table 2:** Self-esteem and general self-efficacy according to previous CBPT exposure Standardized mean differences are expressed as Hedges’ g. Positive values indicate higher scores in the first-listed group. Pairwise p-values remain adjusted using Tukey’s honestly significant difference procedure CBPT: cognitive behavioral play therapy; SD: standard deviation; CI: confidence interval; RSES: Rosenberg Self-Esteem Scale; GSE: General Self-Efficacy Scale

Outcome	CBPT exposure group	N	Mean ± SD	Overall F	Overall p-value	Pairwise comparison	Mean difference	Hedges’ g	95% CI
RSES	No exposure (0 sessions)	50	24.8 ± 3.8	19.28	< 0.001	—	—	—	—
	Limited exposure (1-11 sessions)	40	27.5 ± 3.8			1-11 vs. 0	2.7	0.70	0.28 to 1.13
	Higher exposure (≥ 12 sessions)	35	29.8 ± 3.4			≥ 12 vs. 0	5.0	1.36	0.88 to 1.84
						≥ 12 vs. 1-11	2.3	0.63	0.16 to 1.09
GSE	No exposure (0 sessions)	50	27.1 ± 4.7	17.41	< 0.001	—	—	—	—
	Limited exposure (1-11 sessions)	40	30.0 ± 4.6			1-11 vs. 0	2.9	0.62	0.19 to 1.04
	Higher exposure (≥ 12 sessions)	35	32.9 ± 4.0			≥ 12 vs. 0	5.8	1.30	0.82 to 1.77
						≥ 12 vs. 1-11	2.9	0.66	0.20 to 1.13

The mean RSES score increased progressively across the CBPT exposure groups, from 24.8 ± 3.8 among participants with no previous CBPT exposure to 27.5 ± 3.8 among those who had received 1-11 sessions and 29.8 ± 3.4 among those who had received ≥ 12 sessions. The overall difference between the three groups was statistically significant (F = 19.28, p < 0.001). Tukey-adjusted pairwise comparisons showed significant differences between the no-exposure and 1-11-session groups (mean difference: 2.7; adjusted p = 0.002), between the no-exposure and ≥ 12-session groups (mean difference: 5.0; adjusted p < 0.001), and between the 1-11-session and ≥ 12-session groups (mean difference: 2.3; adjusted p = 0.022).

Similarly, mean GSE scores increased from 27.1 ± 4.7 among participants with no previous CBPT exposure to 30.0 ± 4.6 among those who had received 1-11 sessions and 32.9 ± 4.0 among those who had received ≥ 12 sessions. The overall difference between the groups was statistically significant (F = 17.41, p < 0.001). Tukey-adjusted pairwise comparisons demonstrated significant differences between the no-exposure and 1-11-session groups (mean difference: 2.9; adjusted p = 0.008), between the no-exposure and ≥ 12-session groups (mean difference: 5.8; adjusted p < 0.001), and between the 1-11-session and ≥ 12-session groups (mean difference: 2.9; adjusted p = 0.017).

Correlation analysis

Pearson correlation analysis demonstrated positive correlations between the number of previous CBPT sessions and both self-esteem and general self-efficacy. The number of CBPT sessions was positively correlated with RSES scores (r = 0.48, p < 0.001) and GSE scores (r = 0.46, p < 0.001). Self-esteem and general self-efficacy showed a weak positive correlation (r = 0.21, p = 0.017), indicating a limited association between the two measured outcomes in this sample.

Multivariable analysis

Multivariable linear regression was performed to examine the adjusted associations between the number of previous CBPT sessions and RSES and GSE scores after controlling for age, sex, ADHD status, socioeconomic status, and learning-disability subtype, as shown in Table 3.

**Table 3:** Multivariable linear regression analysis of factors associated with self-esteem and general self-efficacy Analysis: multivariable linear regression adjusted for age, sex, ADHD status, socioeconomic status, and learning-disability subtype. Significance: p < 0.05 RSES: Rosenberg Self-Esteem Scale; CI: confidence interval; GSE: General Self-Efficacy Scale; CBPT: cognitive behavioral play therapy; ADHD: attention-deficit/hyperactivity disorder; R²: coefficient of determination

Predictor	RSES: adjusted β (95% CI)	P-value	GSE: adjusted β (95% CI)	P-value
Number of CBPT sessions	0.28 (0.18 to 0.38)	<0.001	0.34 (0.22 to 0.45)	< 0.001
Age (years)	0.28 (−0.18 to 0.74)	0.230	0.34 (−0.21 to 0.89)	0.223
Female sex vs. male	0.61 (−0.62 to 1.84)	0.329	0.72 (−0.79 to 2.23)	0.348
ADHD, present vs. absent	−1.21 (−2.52 to 0.10)	0.070	−1.48 (−3.01 to 0.05)	0.058
Socioeconomic status	0.74 (0.10 to 1.38)	0.024	0.91 (0.14 to 1.68)	0.021
Learning-disability subtype, overall	—	0.176	—	0.194
Model R²	0.265	—	0.258	—

Following adjustments, the prior numbers of CBPT sessions remained largely relevant to the predetermined indicators. Each additional CBPT session was associated with a 0.28-point increase in the RSES score (adjusted β = 0.28; 95% CI: 0.18-0.38; p < 0.001) and a 0.34-point increase in the GSE score (adjusted β = 0.34; 95% CI: 0.22- 0.45; p < 0.001) based on the fitted regression models. The socioeconomic status turned out to be significantly relevant for the outcomes, too (adjusted β = 0.74; 95% CI: 0.10-1.38; p = 0.024) for RSES and (adjusted β = 0.91; 95% CI: 0.14-1.68; p = 0.021) for GSE. The variables age and sex (i.e., female sex) were not significantly associated with the above indicators. The ADHD variable showed a negative correlation for both RSES (adjusted β = -1.21; 95% CI: -2.52 to 0.10; p = 0.070) and GSE (adjusted β = -1.48; 95% CI: -3.01 to 0.05; p = 0.058), though neither correlation reached significance. The variable of learning disability subtype showed no significance for RSES (p = 0.176) or GSE (p = 0.194). The total RSES model explains 26.5% of the variation in self-esteem variables (R² = 0.265), whereas GSE explains 25.8% of the variation in general self-efficacy (R² = 0.258).

## Discussion

The present study found a significant positive association between previous CBPT exposure and both self-esteem and general self-efficacy among children with learning disabilities. Higher numbers of previous CBPT sessions were associated with higher RSES and GSE scores, and these associations remained statistically significant after adjustment for age, sex, ADHD status, socioeconomic status, and learning-disability subtype. However, given the observational and cross-sectional design of the study, these findings indicate an association rather than a causal relationship between CBPT exposure and psychosocial outcomes.

Previous research has documented psychological difficulties, including concerns related to self-esteem and self-efficacy, among children with learning disabilities [1-5]. This background highlights the importance of examining psychosocial outcomes in this population. The present study contributes to this area of research by examining the association between previous CBPT exposure and self-esteem and general self-efficacy. However, these findings demonstrate associations rather than establish a causal effect of CBPT.

Self-esteem and self-efficacy are important components of psychosocial functioning in children with disabilities. Previous studies have highlighted the importance of addressing the broader psychological and social development of children with learning disabilities rather than focusing exclusively on their academic difficulties [8]. The present findings further support the relevance of assessing psychosocial outcomes alongside academic functioning when evaluating children with learning disabilities.

An important finding of the present study was the progressive increase in RSES scores across the categories of previous CBPT exposure, with the highest scores observed among participants who had received ≥12 sessions. Play-based therapeutic activities may provide children with opportunities to express emotions, address difficulties, and develop problem-solving skills. A systematic review has reported beneficial effects of psychotherapy among children, although the available evidence was insufficient to draw definitive conclusions [20]. The present findings should therefore be interpreted as evidence of an association between greater previous CBPT exposure and higher self-esteem, rather than as evidence that increasing CBPT exposure directly improves self-esteem.

The findings may also be considered in the context of previous studies examining therapeutic interventions among children with behavioral and psychological difficulties. Earlier research has investigated the efficacy of CBPT in children with anxiety-related school phobia and behavioral difficulties [12]. Studies involving children with dyslexia have also reported positive changes in behavioral and problem-solving outcomes following therapeutic interventions [13]. These findings provide a potential context for understanding the positive association between previous CBPT exposure and self-esteem and self-efficacy observed in the present study. However, evidence derived from other clinical or developmental populations may not be directly applicable to children with learning disabilities [21].

The association between CBPT exposure and self-efficacy is also relevant in the context of previous research on self-efficacy among children with specific learning disorders [22]. In the present study, the number of previous CBPT sessions was positively correlated with GSE scores and remained significantly associated with GSE scores after adjustment for the prespecified covariates. RSES and GSE scores were also positively correlated, suggesting that self-esteem and general self-efficacy are related aspects of psychosocial functioning but remain distinct constructs. The use of the GSE in the present study is also consistent with previous research examining its applicability among adolescents [23].

The present analysis also identified a significant association between socioeconomic status and both self-esteem and general self-efficacy. Importantly, the associations between previous CBPT exposure and both outcomes remained statistically significant after adjustment for socioeconomic status and other demographic and clinical variables. Thus, the observed associations were not fully explained by the measured covariates included in the regression models. Nevertheless, residual confounding cannot be excluded. Factors such as family background, severity of learning disability, parental engagement, and availability of rehabilitation services may influence both access to CBPT and psychosocial outcomes and were not fully captured in the present analysis.

Strengths and limitations

The study included a clearly defined population of children with learning disabilities and assessed two relevant psychosocial outcomes, namely self-esteem and general self-efficacy, while adjusting for important demographic and clinical factors. However, the cross-sectional observational design prevents conclusions regarding causality. Previous CBPT exposure may also have varied in intensity and content, and potential confounding factors, such as severity of learning disability and family background, may not have been fully captured. Relying partly on parent/child recall alongside records may have introduced recall or classification error. Finally, the single-institution setting may limit the generalizability of the findings.

## Conclusions

Among children with learning disabilities, previous exposure to CBPT was positively associated with both self-esteem and general self-efficacy. A greater number of previously completed CBPT sessions was associated with higher RSES and GSE scores, and these associations remained statistically significant after adjustment for relevant demographic and clinical variables. However, due to the observational and cross-sectional design, the findings do not establish a causal relationship between CBPT exposure and these outcomes. Further prospective and interventional studies are warranted to evaluate the potential role of CBPT in addressing psychosocial outcomes among children with learning disabilities.

## References

[1] Caqueo-Urízar A, Mena-Chamorro P, Atencio-Quevedo D, Flores J, Urzúa A (2022). Self-esteem in adolescents with learning difficulties: a study from the perspective of the students, parents, and teachers. Psychol Sch.

[2] Martín-Ruiz I, González-Valenzuela MJ, Infante-Cañete L (2023). Emotional adjustment among adolescent students with and without specific learning disabilities. Children (Basel).

[3] Heiman T, Olenik-Shemesh D (2020). Social-emotional profile of children with and without learning disabilities: the relationships with perceived loneliness, self-efficacy and well-being. Int J Environ Res Public Health.

[4] Commodari E, La Rosa VL, Sagone E, Indiana ML (2022). Interpersonal adaptation, self-efficacy, and metacognitive skills in Italian adolescents with specific learning disorders: a cross-sectional study. Eur J Investig Health Psychol Educ.

[5] Sucheta, Das AB (2023). Self-esteem and adaptive behavior in children with specific learning disability. Indian J Clin Psychol.

[6] World Health Organization (2024). Clinical Descriptions and Diagnostic Requirements for ICD-11 Mental, Behavioural and Neurodevelopmental Disorders. https://www.who.int/publications/i/item/9789240077263.

[7] American Psychiatric Association (2022). Diagnostic and Statistical Manual of Mental Disorders, Fifth Edition, Text Revision (DSM-5-TR). https://www.google.com/goto?url=CAESgQEB6zswFRIsZAdBTCgF7ILYqr-7l-nxdZGDeBnVgKf48MrG6BY-SV4tbcsXexMxywvcQTI4XKmhn_CQvsVY_i9n26dvx-SefZJd3-60-J1a36WoDogB7_dBEPdP8vEE7dmzk-bWlDWN6zDmEsHJ129MVtNSTcMcO53KFoovdrv4SlU.

[8] Fehr KK, Russ S, Ievers-Landis C (2016). Treatment of sleep problems in young children: a case series report of a cognitive-behavioral play intervention. Clin Pract Pediatr Psychol.

[9] Massoodi A, Moudi S, Malekiamiri M, Ahangar HG (2024). Comparison of self-esteem and quality of life in 8-12-year-old children with ADHD with and without learning disorders. BMC Psychol.

[10] DuPaul GJ, Gormley MJ, Laracy SD (2013). Comorbidity of LD and ADHD: implications of DSM-5 for assessment and treatment. J Learn Disabil.

[11] Reichenberger V, van der Watt AS, Louw Q, Seedat S, Smythe T (2025). Mapping play-based interventions for children with disabilities in LMICs: a scoping review on cultural relevance, implementation, and impact. EClinicalMedicine.

[12] Shayganfard M, Kaboudi B, Arabsheibani K, Shakiba E, Cheshmekaboudi S (2024). Effectiveness of cognitive-behavioral group play therapy on anxiety-based school refusal and behavioral problems in elementary school boys: a preliminary randomized controlled trial. Arch Psychiatr Nurs.

[13] Vosoughi Kalantari A, Marashian FS, Dasht Bozorgi Z, Hafezi F (2025). Effectiveness of cognitive-behavioral play therapy on behavioral adjustment and problem-solving skills in students with dyslexia. Int J Sch Health.

[14] Shabani M, Sadoughi M, Samkhaniani E (2024). Effectiveness of cognitive-behavioral play therapy on social anxiety and academic self-efficacy in elementary school students with learning disabilities. KMAN Couns Psychol Nexus.

[15] Cheran, Yamini SY (2023). Effectiveness of cognitive behaviour play therapy on level of self esteem among learning disability school age children. J Pharm Negative Results.

[16] Rosenberg M (1965). Society and the Adolescent Self-Image. https://books.google.co.in/books?id=YR3WCgAAQBAJ&printsec=frontcover&redir_esc=y#v=onepage&q&f=false.

[17] Schwarzer R, Jerusalem M (1995). Generalized self-efficacy scale. Measures in Health Psychology: A User's Portfolio. Causal and Control Beliefs.

[18] Moksnes UK, Espnes GA, Eilertsen ME, Bjørnsen HN, Ringdal R, Haugan G (2024). Validation of Rosenberg self-esteem scale among Norwegian adolescents - psychometric properties across samples. BMC Psychol.

[19] Das SK, Philip M, Sudhir PM, Binu VS (2024). Psychometric evaluation of Schwarzer & Jerusalem's General Self-Efficacy Scale among Indian adolescents: a factor analysis and multidimensional item response theory approach. Meas Instrum Soc Sci.

[20] Carrington L, Hale L, Freeman C, Smith D, Perry M (2024). The effectiveness of play as an intervention using International Classification of Functioning outcome measures for children with disabilities - a systematic review and meta-synthesis. Disabil Rehabil.

[21] Çuvadar Baş Y, Karaca S, Ecevi̇t ED (2026). The effects of play-based interventions on social and play skills in children with autism spectrum disorder: a meta-analysis. Ir J Med Sci.

[22] Kiliç Tülü B, Özbek AB, Girli A (2026). A comparative study of self-efficacy, loneliness, and well-being in adolescents diagnosed with autism spectrum disorder and specific learning disorder. J Autism Dev Disord.

[23] Steigen AM, Finbråten HS, Kleppang AL (2022). Using Rasch analysis to assess the psychometric properties of a five-item version of the General Self-Efficacy Scale in adolescents. Int J Environ Res Public Health.

